# Analysis of the Planar Point Identification Accuracy in CMM Measurements

**DOI:** 10.3390/s22187005

**Published:** 2022-09-15

**Authors:** Tomasz Mazur, Lenka Cepova, Tomasz Szymanski, Miroslaw Rucki

**Affiliations:** 1 Faculty of Mechanical Engineering, Kazimierz Pulaski University of Technology and Humanities in Radom, ul. Stasieckiego 54, 26-600 Radom, Poland; 2 Faculty of Mechanical Engineering, VSB-Technical University of Ostrava, 17. listopadu 2172/15, 708 00 Ostrava, Czech Republic; 3 Mitutoyo Polska Sp. z o.o., ul. Graniczna 8A, 54-610 Wrocław, Poland; 4 Institute of Mechanical Science, Vilnius Gediminas Technical University, J. Basanaviciaus Str. 28, LT-03224 Vilnius, Lithuania

**Keywords:** coordinate measuring machine, precision metrology, accuracy, probing point, error minimization

## Abstract

The paper presents the results of the investigations on the direction-dependent accuracy of the point identification during contact probe measurements with a coordinate measuring machine (CMM). Considering the contact point identified by an orthogonal to the surface probe movement, the transformation of coordinates was made in order to calculate the displacement of the measured point. As a result, the positioning accuracy was estimated in three axes. The experiments demonstrated a strong dependence of the displacement on the declination angle. Moreover, it was found that the directional surface texture which provided different roughness in perpendicular directions, had an impact on the positioning accuracy.

## 1. Introduction

Coordinate measuring machines (CMMs) are widely used in industry for three-dimensional measurements of various workpieces [1]. CMMs are capable of determining the spatial coordinates of measurement points that are identified on the surface of a measured object, and then the probed spatial points are calculated to determine the best-fitting geometrical elements [2]. CMMs are regularly calibrated and operate mainly in a stable conditions, and due to the defined ambient conditions ensuring a long-term geometric accuracy, the MPE (Maximum permissible error) parameter can be used to characterize the CMM, according to ISO 10360-1: 2000 and ISO 10360-2: 2009 [3].

In many cases, a non-contact dimensional measurement is desirable, for instance, to perform a continuous measurement during the technological process [4], to analyze complex geometry, especially that achieved in additive manufacturing processes [5], or in the case of soft objects to avoid scratching the surface during the measurement [6]. However, it is widely recognized that the application of tactile probing systems in CMMs are rather favorable because of the higher accuracy obtained, compared to the non-contact ones [7]. Obviously, the knowledge of coordinate measurement principles and the basic rules of CMM operation is crucial for the correct assessment of the accuracy of the obtained results [8]. The single point of uncertainty is very important [9], especially when considering the uncertainty propagation rules. Thus, the present study is focused on the difference between the identified and real contact point in the CMM measurements when the probe ball tip moves at a certain angle toward the measured surface at the moment of contact.

Usually, a stylus used in CMMs for the contact measurement is equipped with a ball tip in order to maintain the same distance from its center in all directions. However, in a complex CMM system, component errors tend to overlap each other, and thus they determine the error vector for every single measurement point [10]. That is why the stylus tip has undoubtedly some impact on the results of the measurements [11]. Similarly, the determination of the contact point between the probe ball tip and the measured surface is very important for the accuracy of measurements [12].

A question on the directional variability of the results obtained from a touch trigger probe remains valid [13]. Johnson et al. [14] described and classified the wide range of factors, which influence the errors of the CMM’s touch trigger probes. The first proposed group consisted of motion-related factors, related to the probe tip’s interaction with the measured surface. The second group consisted of probe errors dependent on the stylus length, mass and rigidity, preload force, etc. In the third group, the included factors generated by the operational mode of operation, while the fifth type of factor was connected with the ambient variations. The last group of factors affecting the probe performance were related measured object factors, addressed in detail by Wozniak and Dobosz [15]. The effect of the direction of probe approach was investigated by Miguel and King [16], who connected it with probe lobing and with kinematic arrangement of the touch trigger probes that caused a variation in trigger force. The differences in accuracy dependent on the type of probe and stylus were confirmed in another study by Sousa [17], which is particularly important considering the huge variety of probing systems designed for different measurement tasks, with different principles and characteristics of tactile probing systems [18]. In the study on the separation of the machine and touch-trigger probe errors [19], it was demonstrated that the latter is dependent on the approach direction. Similarly, the importance of the direction of probe’s approach to the point for running the simulations of the model for CMM’s uncertainty estimation was emphasized [20]. However, these studies did not look for any correlation between the contact point identification and the resulting measurement inaccuracy. The issue was only indicated in the *Good Practice Guide* [21] in terms of the larger variabilities of the results when the movement of the probe was non-perpendicular to the measured surface.

Many studies have been undertaken to perform reliable uncertainty analyses and to improve the accuracy of the CMM measurements [22]. Vrba et al. described the different approaches in the CMM’s uncertainty evaluation [23]. Kubatova et al. [24] performed an analysis of the repeatability and reproducibility of a CMM. Wojtyla et al. [25] proposed a sensitivity analysis, a new method for the uncertainty assessment of coordinate measurements, concerning the dimensions and geometrical deviations on the basis of the MPE. Stojadinovic et al. [26] made a new contribution to the development of the digital twin to support an inspection strategy of the CMM, by configuring a virtual CMM. Ren et al. worked out a novel method based on the classical Abbé principle to measure the parameter error of the trigger probe to introduce the compensation during the measurement process [27].

In the present study, the effect of several parameters on the accuracy of the planar point identification on the measured surface was examined. These were: direction of the point contact determined by the angle β, probe ball tip diameter, and the roughness of the measured surface. The identification accuracy was analyzed in terms of the position accuracy (AP) vector [28]. The AP parameter was used according to the definition given in the standard PN-ISO 9283: 2003, and found to be very useful in the case of the accuracy analysis of industrial robots [29]. This issue was undertaken because it is not always possible to reach a probing point with the perpendicular movement of the probe ball tip toward the measured surface, as it was described in [30]. Moreover, sometimes quick or initial measurements are performed without keeping the exact perpendicularity movement of the ball tip. Obviously, this sort of measurement would introduce certain geometrical and computational errors, thus giving inaccurate results for the coordinate dimensional measurements. The practice indicates that the higher the deviation is from the perpendicularity, the larger the error will be. The error is further increased when the larger diameters of the probe ball tip are used. Trying to minimize the impact of the non-perpendicularity of the probe movement, some solutions have been proposed, such as the Mitutoyo MCOSMOS CAT1000 module capable of calculating each time anew the direction measurement of the probing point, based on a 3D model of the complex surface. It is important to keep the consistency in the CMM measurement procedure [31], however, the issue of the non-perpendicular movement of a probe has not been properly addressed and the quantitative recommendations are unavailable, especially in terms of the larger declinations from the perpendicularity.

## 2. Materials and Methods

### 2.1. Measurement Devices and Procedures

In the planar point identification error analysis, the option was chosen, making it possible to set the probe ball tip movement to the same contact point from different directions. However, the axis of the stylus was always positioned vertically, i.e., the angles between it and the coordinate axes were *β_x_ = β_y_* = 0°.

**Figure 1:**
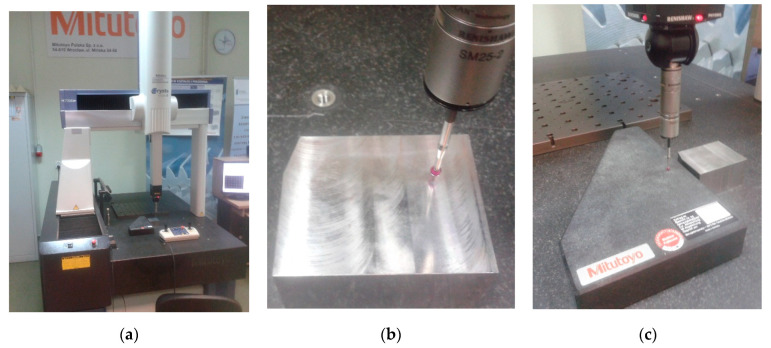
Experimental setup: (**a**) CMM Mitutoyo Crysta-Apex C7106; (**b**) Sample 1, steel machined by face milling; (**c**) Sample 2, granite standard of a right angle made by Mitutoyo.

The experiments were carried out using a coordinate measuring machine CNC Mitutoyo Crysta-Apex C7106, as shown in Figure 1a. With the application of the measurement head Renishaw PH10MQ, its maximum permissible error was *MPE_E_* = 1.7 + 0.3*L*/100 [μm]. In the measurement experiments, two scanning probes of the SP-25M type were used, one denoted as ‘Probe 1’ had a ball tip with a 2 mm diameter and a stylus holder length of 173.35 mm, while the other denoted as ‘Probe 2’ had a ball tip with a 5 mm diameter and a stylus holder length of 169.35 mm. Based on the previous experience, these two diameters were found to be the most commonly used.

Using the control software MCOSMOS 3.2 R.13, the CNC movement parameters were defined as follows:

1. High precision measurement at the speed: *v* = 3 mm/s;
2. Safety distance: 0.5 mm;
3. Pretravel distance: *a* = 5 mm;
4. Probe deflection value: 0.3 mm.

For the experiments, two different surfaces were chosen. One was made out of steel machined by face milling (Sample 1 shown in Figure 1b), and other was a granite standard of a right angle made by Mitutoyo (Sample 2 shown in Figure 1c). The surface roughness of both samples was measured in the area of the destined contact with the probe ball tip. The directions of roughness measurement along the probe tip movement was denoted as α = 0° and perpendicular to it as α = 90°. The roughness was measured with a Mitutoyo SJ-500P profilometer with a diamond stylus at the following parameters: evaluation length 4 mm, sampling length 0.8 mm, and respective cut-offs *λ_c_* = 0.8 mm and *λ_s_* = 0.0025 mm. The parameter *Ra* was measured eight times in two directions corresponding with the probe movement α = 0° and α = 90°, four times in each direction. The respective *Ra* values are shown in Table 1.

The experiments were performed with the ball tip moving in two perpendicular directions, at α = 0° and α = 90°, defined by the main plane *x-y* of the local coordinate system corresponding with the upper surface of the measured sample. At each sample surface, 72 repetitions were made in the CNC mode in eight probing points placed evenly on the sample surface, forming a circle with a 10 mm diameter. Following the calculation of the center of this circle, it was set as an initial point *A* of the coordinate system *xyz* and used in the experiments as a contact point to check its identification. Its position in the CMM’s coordinate system, was determined as *X* = 410, *Y* = 410, and *Z* = 184 mm. The direction of *x*-axis was chosen corresponding with the direction of maximal roughness defined along the probe movement at α = 0°. The respective *y*-axis was set in the direction of minimal roughness at α = 90°, i.e., perpendicular to the probe movement.

**Figure 2:**
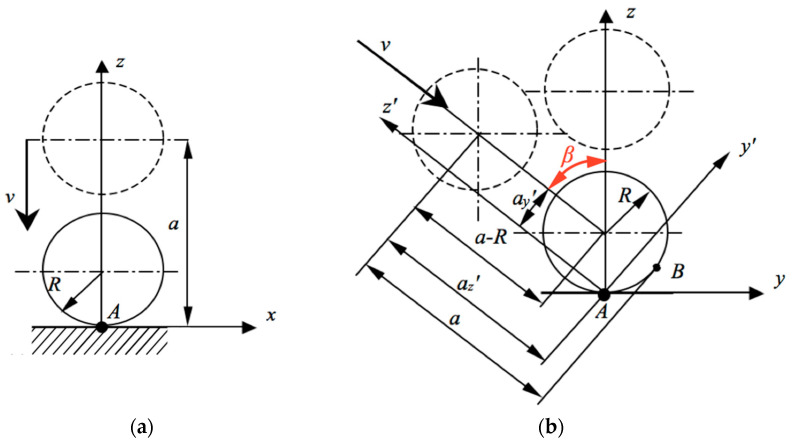
Contact point *A* between the ball tip and the measured surface: (**a**) Basic coordinate system *oxyz* for the identification of contact point *A*; (**b**) Coordinate system after the rotation of the local coordinate system by angle *β*.

In the abovementioned system, shown in Figure 2a, the movement of the probe ball tip of radius *R* toward the measured surface is perpendicular, along the *z*-axis, with angle *β_x_* = 0°. The stylus movement velocity is *v*. The distance *a* between the contact point *A* and the center of the ball tip had to be constant for all measurements in order to minimize the effect of the probe pretravel [32], so the starting point it was set at *a* = 5 mm. This way, point *A* was made a reference point for the rest of the measurements with different movement angles.

However, if the direction of the stylus movement *v* is not perpendicular to the measured surface, the system identifies point *B*, while the real contact took place in point *A*, as shown in Figure 2, thus generating an error. In order to analyze this error and ensuring the same position of the probing point irrespective of the movement direction, the CNC options were used as follows. The coordinate system was rotated accordingly, so that the starting point of the movement laid on the newly defined *z′*-axis. Then, movement *v* took place along the *z′*-axis down to the same contact point *A*, as it is shown in Figure 2b.

Geometrically, when the angle between the *z*-axis and *z′*-axis is *β* ≠ 0°, the starting point must undergo some correction. The center of the ball tip along the axis *ox′* will be changed from 0 to *a_y_′* = +*R* × sin*β*, where *R* is the radius of the ball tip. Moreover, to keep the same travel length *a* − *R* of the ball tip center, coordinate *z′* should be corrected, too. The coordinate of the starting point along the *oz′* axis should be reduced from the basic value *z* = 5 mm at *β* = 0° down to the value *a_z_′* = 5 − *R* × (1 − cos*β*). Following the corrections of the starting point in the declined coordinate system *ox′y′z′*, the contact point *A* on the planar surface remained the same, while the touching points on the probe ball tip changed. In addition, the travel between the starting point and touch point *A* was always close to the assumed value 5 − *R* mm.

Analysis of the measurement results with different angles of the probe ball tip movement required relevant equations to recalculate the coordinates appropriately.

### 2.2. Computations

**Figure 3:**
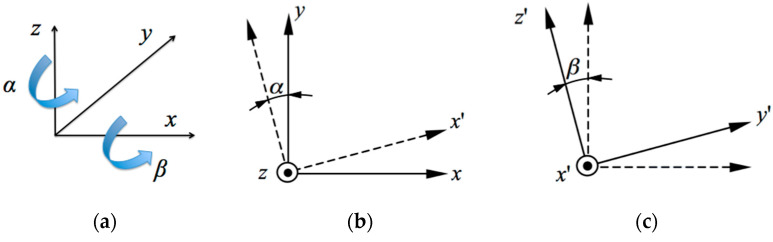
Transformations of the basic coordinate system *oxyz* to the declined coordinates: (**a**) Rotation directions; (**b**) Rotation around the *z*-axis by the angle α; (**c**) Rotation around the *x*′-axis by the angle *β*.

It was necessary to prepare the equations able to compare the registered coordinates of the probing points at the perpendicular and declined movements of the probe ball tip to the measured surface. In particular, the equations had to describe the transformation of the coordinates from the *ox′y′z′* system to *oxyz* after two subsequent rotations. The first rotation was around the *z*-axis by angle α, and the second rotation around the *x*-axis by angle *β*, as it is shown in Figure 3.

Thus, the first partial transformation, as shown in Figure 3b, can be described by the matrix *A_z_*, as follows [28]:

(1) $$A_{z}=\left[\begin{array}{ccc} \cos\alpha & \sin\alpha & 0\\ -\sin\alpha & \cos\alpha & 0\\ 0 & 0 & 1 \end{array}\right].$$

The second partial transformation, as shown in Figure 3c, corresponds with the matrix *A_x_*, as follows:

(2) $$A_{x}=\left[\begin{array}{ccc} 1 & 0 & 0\\ 0 & \cos\beta & \sin\beta \\ 0 & -\sin\beta & \cos\beta \end{array}\right].$$

Then, according to the abovementioned procedure with the two subsequent rotations, the matrix *A_zx_* represents the transformation, as follows:

(3) $$A_{zx}=\left[A_{x}\cdot A_{z}\right]=\left[\begin{array}{ccc} \cos\alpha & \sin\alpha & 0\\ -\sin\alpha \cos\beta & \cos\alpha \cos\beta & \sin\beta \\ \sin\alpha \sin\beta & -\cos\alpha \sin\beta & \cos\beta \end{array}\right].$$

When the coordinates of the point [*x′, y′, z′*] have to be transformed to the basic coordinate system *oxyz,* as shown in Figure 1, the following equation can be applied:

(4) $$\left[\begin{array}{l} x\\ y\\ z \end{array}\right]=\left[A_{x}\cdot A_{z}\right]{\;}^{T}\left[\begin{array}{l} x\prime \\ y\prime \\ z\prime \end{array}\right]=\left[\begin{array}{ccc} \cos\alpha & -\sin\alpha \cos\beta & \sin\alpha \sin\beta \\ \sin\alpha & \cos\alpha \cos\beta & -\cos\alpha \sin\beta \\ 0 & \sin\beta & \cos\beta \end{array}\right]\left[\begin{array}{l} x\prime \\ y\prime \\ z\prime \end{array}\right]$$

to derive the final coordinates as follows:

*x* = *x′* cos*α* − *y′* sin*α* cos*β* + *z′* sin*α* sin*β*, (5)

*y* = *x′* sin*α* + *y′* cos*α* cos*β* − *z′* cos*α* sin*β*, (6)

*z* = *y′* sin*β* + *z′* cos*β*. (7)

### 2.3. Test Campaign

For the experiments and calculations, the following values of angle α were chosen: 0°, 45°, 90°, 135°, 180°, 225°, 270°, and 315°. Four of the angles corresponded with the directions of maximal and minimal roughness of the measured surface, namely, α = 0° and α = 180° with *Ra*_max_, and α = 90° and α = 270° with *Ra*_min_. Considering the possible influence of the probing system, the angles between 180° and 360° allowed for checking the symmetry of the generated errors.

The declinations of the *z′*-axis were used as follows. First, the basic measurement was made at *β* = 0°, i.e., with ball tip movement *v* perpendicular to the measured surface (Figure 1). Next, the declination *β* took the values, as follows: 2°, 4°, 8°, 12°, 16°, 20°, 30°, and 40°. Each measurement was repeated 25 times.

Accordingly, for eight values of angle α and nine values of angle *β*, the number of the results was 8 × 9 × 25 = 1800 for one probe using one sample surface. Having two probes and two samples to be measured, the overall number of the obtained results for the analysis was 7200.

## 3. Results and Discussion

### 3.1. Identification of the Probing Points

**Figure 4:**
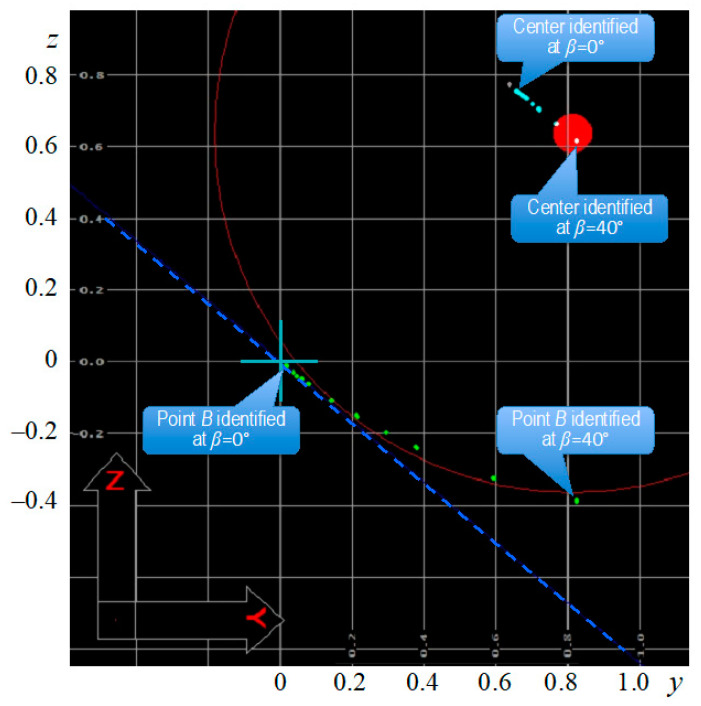
The screen with results after 225 measurements of the point *B* at *α* = 90° and nine different values of *β*, with Sample 1, Probe 1.

Figure 4 presents the example of the screen displayed during the measurement, i.e., the identification of point *B* as shown in Figure 2. In that case, all of the results were obtained at α = 90° from the steel sample (Sample 1) with Probe 1. This angle corresponded with the direction of the smallest roughness, as it was indicated in Table 1. The ball tip profile is marked with a thin red curve, while light blue points corresponding with the ball tip center positions identified at different movement angles *β* from 0° to 40°. For the angle combinations *α* = 90° and *β* = 40°, the basic plane is shown by the dark blue dotted line in the starting coordinate system *oxyz* observed along the *ox*-axis, declined to the *oz*-axis by the 40° angle. The green points represent the coordinates of the identified contacts between the probe ball tip and the measured surface.

It can be seen that the coordinates of the ball tip center moved in the direction parallel to the basic plane, along the probe ball tip movement vector, with an increasing *β* angle. A similar trend can be seen among the green points corresponding with the identified contact points. The first, placed most closely to the point with the coordinates (0, 0, 0), is the basic point that is the result of 25 repetitions at *α* = 90° and *β* = 0°. The differences between the repetitions are negligibly small and not seen at this magnification. To illustrate this, the mean values of the obtained and recalculated coordinates are collected in Table 2 together with the values of ±3*s* ranges. The latter ones varied from 2.12 × 10^−17^ up to 0.003 mm, which was close to the maximum permissible error *MPE_E_* = 1.7 + 0.3*L*/100 μm, and thus negligible.

Other green points in Figure 4 represent the groups of the results for angles *β* ≠ 0°, so that the largest angle *β* = 40° of the ball tip movement vector *v* declination from the perpendicular provided the most distant identification from the real point *B*. The trend is very clear, the larger the angle *β* of the coordinate system *ox′y′z′*, the farther the point identified by CMM from the real one will be.

**Figure 5:**
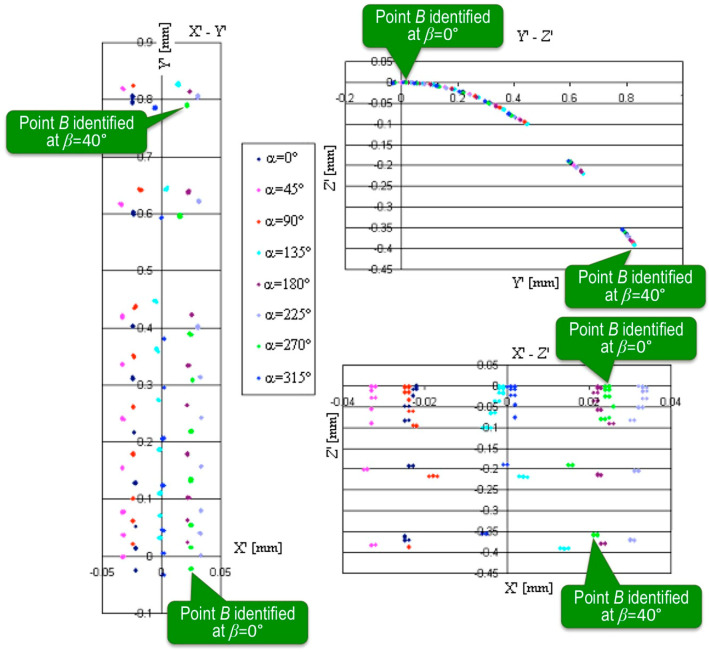
The diagrams *x′-y′*, *y′-z′*, and *x′-z′* of the measurement results of point *B* on the steel surface (Sample 1) at different angles *α* and *β*.

**Figure 6:**
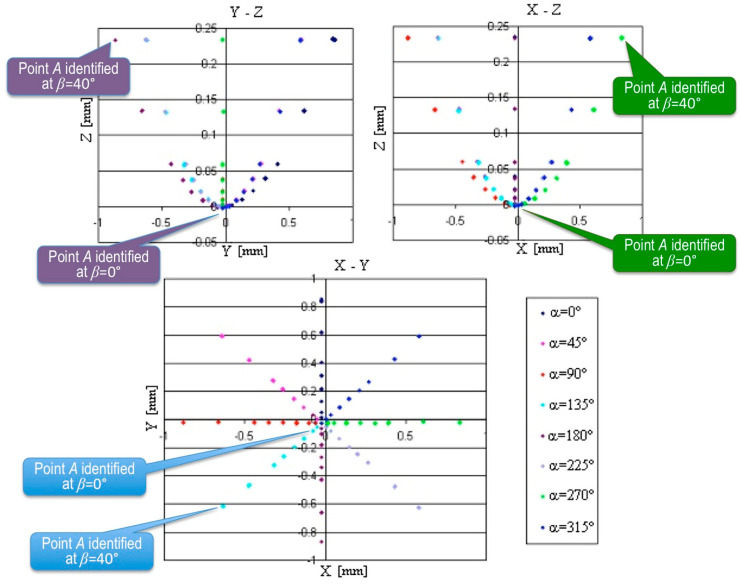
The diagrams *x-y*, *y-z*, and *x-z* of measurement results of point *A* on the steel surface (Sample 1) at different angles *α* and *β*.

The full set of the results collected from Sample 1 made out of steel using Probe 1 with a 2 mm ball tip diameter is shown in Figure 5 in the form of diagrams in the respective coordinate planes *x′-y′*, *y′-z′*, and *x′-z′*. The travel of the ball before contact was 5 mm. The different colors correspond with the different values of *α* angle, and each point represents 25 repetitions with almost similar results. The diagrams of the recalculated coordinates of point *A* are shown in Figure 6.

In both Figure 5 and Figure 6, the points of the same color close to the center of coordinate system (0,0,0) were obtained at *β* = 0°, while the most distant ones were obtained at *β* = 40°. In the diagrams of Figure 5, the respective callouts were placed near the green points obtained at *α* = 270°, but the pattern was identical for each value of *α*. Similarly, in Figure 6, the callouts were given to distinguish the points obtained for different values of angle *β*. As every step of the declination angle *β* increased, the group of the results found its place farther from the central point (0,0,0). Thus, the identification errors of points *A* and *B* increased, accordingly.

From the diagram *x-y* in Figure 6, it can be concluded that all of the coordinates are shifted from the basic point according to the direction of the probe movement. However, the displacements are not steady. When the movement took place along the highest roughness direction at *α* = 90° and *α* = 270°, the smallest displacement was detected, while the angles *α* = 0° and *α* = 180° corresponding with the smallest roughness provided the largest displacements. It is noteworthy that at the small declination angles *β* recalculated the points laid in the plane *x-y*, i.e., on the measured surface. However, increase of the angle *β* caused the increase of the *z* coordinate, moving the identified points away from contact with the real surface.

### 3.2. Positioning Accuracy Analysis

Unfortunately, the standard ISO 10360-2:2001 does not identify the accuracy of the probing point. Thus, in order to assess the positioning accuracy, the parameter *AP* was taken according to the standard PN-ISO 9283:2003. Even though the latter standard was withdrawn in 2012, there have been no other proposed definitions for the position accuracy (*AP*). In the ISO 9283, the *AP* was defined as a deviation between the theoretical (set) value of the position and the average of the real positions from *n* repetitions performed from the same direction. Moreover, the multidirectional positioning accuracy was defined using *n* repetitions performed from three orthogonal directions.

We found it useful to adopt the abovementioned definitions to the conditions of our research. The average from 8 × 25 = 200 repetitions at angle *β* = 0° was taken as the set value of the basic point *A*. Similarly, the points obtained from the non-perpendicular movement of the probe, were identified in 64 directions for *α* ≥ 0° and *β* > 0°. For these results, the vector *AP* defined by the components *AP_x_*, *AP_y_*, *AP_z_*, was determined as follows:

(8) $$\begin{array}{cc} AP=\sqrt{AP_{x}^{2}+AP_{y}^{2}+AP_{z}^{2}} & =\sqrt{{\left(\frac{1}{25}\overset{25}{\underset{i=1}{\sum }}x_{i}-x_{c}\right)}^{2}+{\left(\frac{1}{25}\overset{25}{\underset{i=1}{\sum }}y_{i}-y\right)}^{2}+{\left(\frac{1}{25}\overset{25}{\underset{i=1}{\sum }}z_{i}-z\right)}^{2}} \end{array}$$

where *x_c_*, *y_c_*, *z_c_* are coordinates of the basic point *A* obtained at *β* = 0°; *x_i_*, *y_i_*, *z_i_* are coordinates of the actually measured probing point; and *n* = 25 is the number of repetitions.

The results of the positioning accuracy calculations for steel (Sample 1 and Probe 1) in two directions *α* = 0° (maximal roughness) and *α* = 90° (minimal roughness) are shown in Table 3.

When the probe movement direction at *α* = 0° is along the maximal roughness, the respective coefficient *AP_y_* describes the deviation of the identified points collected along the *y*-axis. When the system is rotated by *α* = 90°, the maximal roughness direction is connected with the axis *ox′*, while the probe moves along the minimal roughness direction corresponding with *y′*-axis. When the coordinate system is recalculated back to the initial one, the coefficient *AP_x_* describes the deviation along the axis of the minimal roughness. For easy comparison, these values of *AP_y_* for *α* = 0° and *AP_x_* for *α* = 90° are emphasized in bold in Table 3. The absolute values of the *AP* along the maximal roughness, i.e., *AP_y_* for *α* = 0°, are higher than that of *AP_x_* for *α* = 90° at smaller values of *β* = 2°, *β* = 4°, *β* = 8°, but for the larger angles *β,* they are lower.

### 3.3. Effect of the Probe Diameter

**Figure 7:**
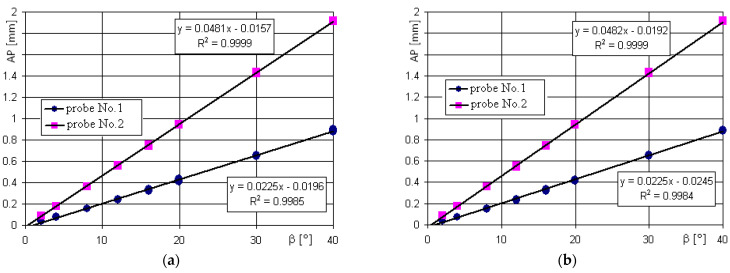
Positioning accuracy *AP* at different angles *β* for Probe 1 (∅2 mm) and Probe 2 (∅5 mm). The measured samples were: (**a**) Sample 1 out of steel; (**b**) Sample 2 out of granite.

**Figure 8:**
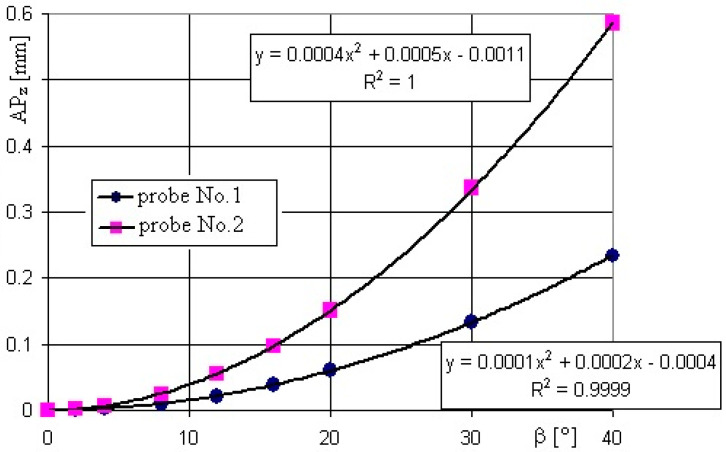
Positioning accuracy along the *z*-axis *AP_z_* = *f* (*β*) for Probe 1 and Probe 2, Sample 1.

For both Samples 1 and 2, the steel and granite, the effect of the probe diameters on the point identification was the same. As illustrated by Figure 7, where the positioning accuracy *AP* is almost the same for the steel and granite samples. An example of the positioning accuracy results along the *z*-axis *AP_z_* = *f* (*β*) are shown in the diagram in Figure 8. An initial attempt of the normalization of the results indicated that the final normalized function appeared to be less clear and rather useless, so it was decided to present the results as they were obtained.

It is noteworthy that the *AP* is increasing proportionally with the probe movement angle *β*. The values of the *AP* are always positive, and for the probe ball tip with a ∅5 mm, they are ca. 2 times larger than that for the probe ball tip with a ∅2 mm. At the same time, the differences between the steel surface with a smaller roughness and granite surface with a greater roughness are too small to be noticed at this scale. For this reason, the effect of the probe diameter will be analyzed using the example of Sample 1 only.

**Figure 9:**
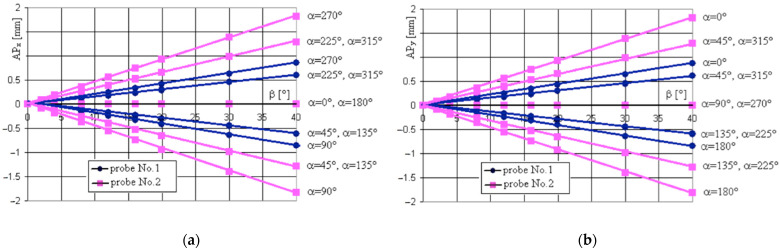
Values of the positioning accuracy *AP* components at different angles *β* for Probe 1 and Probe 2, Sample 1: (**a**) *AP_x_ = f* (*β*); (**b**) *AP_y_ = f* (*β*).

Some difference can be seen in the positioning accuracy along the *z*-axis, as shown in Figure 8 and the *AP* along the axes *x* and *y* presented in Figure 9a,b. The values of *AP_z_* are always positive and again, more than 100% larger for Probe 2 with a ball tip diameter of ∅5 mm for each respective probe movement angle *β*.

In the case of function *AP_z_ = f (β), as* shown in Figure 8, the second order curves can be applied in the investigated range of values. In turn, the dependencies of components *AP_x_* and *AP_y_* on angle *β* are linear in the investigated range. For the different directions described by *α*, *AP_x_* and *AP_y_* are positive (+) or negative (−) with similar absolute values for the respective angles *α*, revealing a strong effect of the roughness of the measured surface. It is seen that the larger diameter of Probe 2 provided larger positioning errors. The largest values of the *AP_x_* corresponded with angles *α* = 90° and *α* = 270° (minimal roughness), as seen in Figure 9a, are similar to the ones of the *AP_y_* component for angles *α* = 0° and *α* = 180° (maximal roughness), as shown in Figure 9b. The increase of the probe ball tip diameter from ∅2 mm up to ∅5 mm always resulted with the positioning error increase by more than 100%.

### 3.4. Effect of the Measured Surface

**Figure 10:**
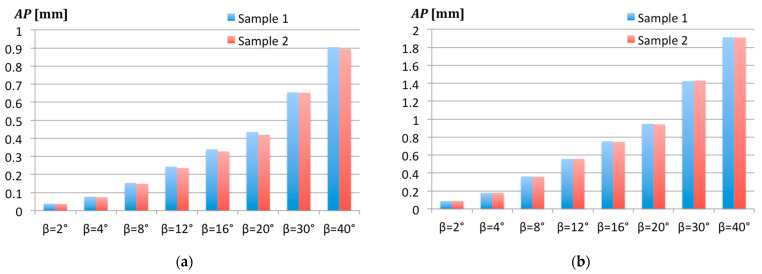
Measured surface impact on the positioning accuracy *AP* at different angles *β* for *α* = 0°: (**a**) For Probe 1 (∅2 mm); (**b**) For Probe 2 (∅5 mm).

It is obvious that each surface has its own peculiarities dependent on many factors. In our study, we wanted to check if two different materials with a different roughness have any impact on the results, when the probe movement is non-perpendicular to the measured surface. Figure 10 presents the positioning accuracy results for two samples, steel (Sample 1) and granite (Sample 2), measured for *α* = 0° in the direction of the highest roughness *Ra*.

In the graphs, the distinguishable differences between the steel and granite samples appear at the probe movement declinations *β* = 12°, *β* = 16°, and *β* = 20° for the smaller Probe 1, and only at *β* = 16° for the larger Probe 2. In the latter case, the difference between the *AP* values for Sample 1 and Sample 2 at *β* = 16° was 7.6 μm. Thus, it may be concluded that the roughness is the main contributor among the surface material characteristics since its effect is largely reduced by the increased diameter of the probe ball tip.

Moreover, some interesting observations can be made from the analysis of the components *AP_x_*, *AP_y_*, and *AP_z_*. Their values are collected in Table 4 and Table 5.

The large majority of the *AP_y_* values are smaller for Sample 2, as marked in bold in Table 4 and Table 5. This direction corresponds with the *y*-axis in the direction of *α* = 0°. Only in one case, the points identified on the granite surface of Sample 2 had substantially higher *AP_y_* values than that of steel surface of Sample 1. It took place at *β* = 30°. It may be assumed that this component was decisive in most cases, whether the *AP* was higher for the granite surface or the steel surface. In all points, this component was at least several times larger than *AP_x_* and *AP_z_*.

## 4. Conclusions

The measurements and calculations performed in this study provided simple and exact equations that describe how accurately the measuring point identification on the plane surface is when the probe’s movement is non-perpendicular to it. It was demonstrated that the probing point might be identified with an error as large as 0.110 mm when the declination was as small as *β* = 4°, while the maximum permissible error was *MPE_E_* ≈ 2 μm. The larger angles, *β* > 20° in the performed experiments, can increase this error above 1 mm.

The research indicated the absolute necessity for keeping the perpendicular direction of the probe movement toward the measured surface when collecting a probing point. Only under this condition, the options “compensated point” and “point on the material” can truly provide the coordinates of the probing point on the measured surface. Otherwise, the systematic error generated by the non-perpendicular movement of the probe should be corrected accordingly. It should be carried out in any measurement, where it is impossible to ensure the perpendicular direction of the probe movement.

The parameter of the positioning accuracy *AP* was found useful for the assessment of the point identification during the CMM measurement. It indicated a strong effect of the probe diameters on the point identification. In the experiments, the probe ball tip with a diameter of ∅2 mm generated two times larger the direction-dependent errors than the one with a diameter of ∅5 mm.

Moreover, very small differences in the *AP* values were found between the respective results obtained for the steel and granite surfaces. From the initial results it may be assumed that the impact of the material on the direction-dependent errors is negligible, but each individual task may reveal its own peculiarities in terms of roughness, friction, etc.

The results revealed the unequivocal symmetry of the coefficients *AP_x_* and *AP_y_* related to the plane *x-y*, in terms of the average values for the auxiliary directions (α = 45°–α = 135° and α = 225°–α = 315°). In both cases, the values are close to the ones obtained along the axes *x* and *y*, where one of the components was dominating while the other was close to zero.

## Figures and Tables

**Table 1:** Roughness of the sample surfaces in the two directions of measurement.

Direction	Angle *α*	Sample 1	Sample 2
Along the probe movement	0°	*Ra* = 0.8 μm	*Ra* = 1.7 μm
Perpendicular to the probe movement	90°	*Ra* = 0.3 μm	*Ra* = 0.7 μm

**Table 2:** Coordinates of the obtained *x′y′z′* and recalculated *xyz* coordinates of the respective points *B* and *A*, for Sample 1, Probe 1, *α* = 90°.

		*β* = 0° (Reference)	*β* = 2°	*β* = 4°	*β* = 8°	*β* = 12°	*β* = 16°	*β* = 20°	*β* = 30°	*β* = 40°
Point *B*	*x′*	−0.0242	−0.0242	−0.0240	−0.0241	−0.0240	−0.0231	−0.0220	−0.0180	−0.0240
	*y′*	0.0220	0.0614	0.1008	0.1793	0.2610	0.3500	0.4370	0.6416	0.8249
	*z′*	−0.0002	−0.0020	−0.0048	−0.0153	−0.0332	−0.0603	−0.0951	−0.2167	−0.3870
Standard deviations	±3*s_x′_*	0.001225	0.001308	0.0006	0.000995	2.12 × 10^−17^	0.000831	0.0006	0.001054	2.12 × 10^−17^
	±3*s_y′_*	2.12 × 10^−17^	0.00147	0.001873	0.003089	0.002522	0.002666	0.002804	0.002121	0.001578
	±3*s_z′_*	0.000408	0.0002	0.000374	0.000476	0.000436	0.000476	0.0004	0.000476	0.000455
Pont *A*	*x*	−0.0220	−0.0614	−0.1009	−0.1797	−0.2622	−0.3531	−0.4432	−0.6640	−0.8806
	*y*	−0.0242	−0.0242	−0.0240	−0.0241	−0.0240	−0.0231	−0.0220	−0.0180	−0.0240
	*z*	−0.0002	0.0001	0.0022	0.0098	0.0218	0.0386	0.0601	0.1331	0.2338

**Table 3:** Positioning accuracy *AP* [mm] analysis for Sample 1, Probe 1.

		*β* = 2°	*β* = 4°	*β* = 8°	*β* = 12°	*β* = 16°	*β* = 20°	*β* = 30°	*β* = 40°
*α* = 0°	*AP_x_*	0.0000	0.0000	−0.0001	−0.0010	−0.0024	−0.0025	−0.0013	−0.0029
	*AP_y_*	**0.0381**	**0.0767**	**0.1526**	**0.2420**	**0.3366**	**0.4311**	**0.6410**	**0.8742**
	*AP_z_*	0.0006	0.0024	0.0100	0.0225	0.0393	0.0598	0.1343	0.2343
	*AP*	0.0381	0.0767	0.1529	0.2430	0.3389	0.4352	0.6549	0.9051
*α* = 90°	*AP_x_*	−**0.0394**	−**0.0789**	−**0.1577**	−**0.2402**	−**0.3311**	−**0.4212**	−**0.6420**	−**0.8586**
	*AP_y_*	0.0000	0.0002	0.0001	0.0002	0.0011	0.0022	0.0062	0.0002
	*AP_z_*	0.0003	0.0024	0.0100	0.0220	0.0388	0.0603	0.1333	0.2340
	*AP*	0.0394	0.0789	0.1580	0.2412	0.3334	0.4255	0.6557	0.8899

**Table 4:** Positioning accuracy *AP* [mm] components for Probe 1 (∅2 mm).

		*β* = 2°	*β* = 4°	*β* = 8°	*β* = 12°	*β* = 16°	*β* = 20°	*β* = 30°	*β* = 40°
Sample 1	*AP_x_*	0.0000	0.0000	−0.0001	−0.001	−0.0024	−0.0025	−0.0013	−0.0029
	*AP_y_*	0.0381	0.0767	0.1526	0.2420	0.3366	0.4311	0.6409	0.8742
	*AP_z_*	0.0006	0.0024	0.0100	0.0225	0.0393	0.0598	0.1343	0.2344
	*AP*	0.0381	0.0767	0.1529	0.243	0.3389	0.4352	0.6549	0.9051
Sample 2	*AP_x_*	0.0001	0.0000	−0.0004	−0.0007	−0.0018	−0.0027	−0.0037	−0.0036
	*AP_y_*	**0.0368**	**0.0741**	**0.1481**	**0.2349**	**0.3251**	**0.4152**	**0.6387**	**0.8633**
	*AP_z_*	0.0006	0.0025	0.0096	0.0218	0.0391	0.0606	0.1348	0.235
	*AP*	**0.0368**	**0.0741**	**0.1484**	**0.2359**	**0.3274**	**0.4196**	**0.6527**	**0.8948**

**Table 5:** Positioning accuracy *AP* [mm] components for Probe 2 (∅5 mm).

		*β* = 2°	*β* = 4°	*β* = 8°	*β* = 12°	*β* = 16°	*β* = 20°	*β* = 30°	*β* = 40°
Sample 1	*AP_x_*	0.0000	−0.0002	−0.0006	−0.0022	−0.0033	−0.0042	−0.0012	−0.0032
	*AP_y_*	0.0889	0.1784	0.3610	0.5544	0.7474	0.9355	1.3853	1.8211
	*AP_z_*	0.0013	0.0060	0.0243	0.0551	0.0976	0.1512	0.3365	0.5876
	*AP*	0.0889	0.1785	0.3618	0.5571	0.7538	0.9477	1.4256	1.9136
Sample 2	*AP_x_*	−0.0004	−0.0001	0.0001	0.0001	0.0001	0.0002	0.0001	0.0001
	*AP_y_*	0.0897	0.1785	**0.3581**	**0.5535**	**0.7399**	**0.9300**	1.3897	**1.819**
	*AP_z_*	0.0013	0.0059	0.0238	0.0543	0.0967	0.1506	0.3349	0.5848
	*AP*	0.0897	0.1786	**0.3589**	**0.5561**	**0.7462**	**0.9421**	1.4295	**1.9107**

## Data Availability

Data available on request.

## References

[1] Sato O., Takatsuji T., Miura Y., Nakanishi S. (2021). GD&T task specific measurement uncertainty evaluation for manufacturing floor. Meas. Sens..

[2] Savio E., Chatti S., Laperrière L., Reinhart G., Tolio T. (2019). Coordinate Measuring Machine. CIRP Encyclopedia of Production Engineering.

[3] Blecha P., Holub M., Marek T., Jankovych R., Misun F., Smolik J., Machalka M. (2022). Capability of measurement with a touch probe on CNC machine tools. Measurement.

[4] Rucki M. (2020). Dynamics of in-process control with non-contact air gauges. Rep. Mech. Eng..

[5] Piratelli-Filho A., Souza P.H.J., Arencibia R., Anwer N. (2014). Study of Contact and Non-contact Measurement Techniques Applied to Reverse Engineering of Complex Freeform Parts. Int. J. Mech. Eng. Autom..

[6] Yu W., Zhu X., Mao Z., Liu W. (2021). The Research on the Measurement System of Target Dimension Based on Digital Image. J. Phys. Conf. Ser..

[7] Kaťuch P., Dovica M., Slosarčik S., Kováč J. (2012). Comparision of Contact and Contactless Measuring Methods for Form Evaluation. Procedia Eng..

[8] Kopáčik A., Erdélyi J., Kyrinovič P. (2020). Engineering Surveys for Industry.

[9] Müller A.M., Hausotte T. (2020). Determination of the single point precision associated with tactile gear measurements in scanning mode. J. Sens. Sens. Syst..

[10] Sładek J.A. (2016). Coordinate Metrology.

[11] Zelinka J., Čepová L., Gapiński B., Čep R., Mizera O., Hrubý R., Diering M., Wieczorowski M., Brown C. (2020). The Effect of a Stylus Tip on Roundness Deviation with Different Roughness. Advances in Manufacturing II. Manufacturing 2019.

[12] Ito S., Tsutsumi D., Kamiya K., Matsumoto K., Kawasegi N. (2020). Measurement of form error of a probe tip ball for coordinate measuring machine (CMM) using a rotating reference sphere. Precis. Eng..

[13] Aston R.A.E., Davis J., Stout K.J. (1997). A probing question: A customer’s investigation into the directional variability of a coordinate measuring machine touch trigger probe. Int. J. Mach. Tools Manuf..

[14] Johnson R.P., Yang Q., Butler C. (1998). Dynamic error characteristics of touch trigger probes fitted to coordinate measuring machines. IEEE Trans. Instrum. Meas..

[15] Woźniak A., Dobosz M. (2005). Influence of measured objects parameters on CMM touch trigger probe accuracy of probing. Precis. Eng..

[16] Miguel P.C., King T., Abackerli Á. (1998). A review on methods for probe performance verification. Measurement.

[17] Sousa A.R. (2018). Metrological evaluation of a Coordinate Measuring Machine with 5-axis measurement technology. Procedia CIRP.

[18] Weckenmann A., Estler T., Peggs G., McMurtry D. (2004). Probing Systems in Dimensional Metrology. CIRP Ann..

[19] Nafi A., Mayer J.R.R., Wozniak A. (2011). Novel CMM-based implementation of the multi-step method for the separation of machine and probe errors. Precis. Eng..

[20] Gąska P., Gąska A., Sładek J., Jędrzejewski J. (2019). Simulation model for uncertainty estimation of measurements performed on five-axis measuring systems. Int. J. Adv. Manuf. Technol..

[21] Flack D. (2014). Measurement Good Practice Guide No. 43: CMM Probing.

[22] Cheng Y., Wang Z., Chen X., Li Y., Li H., Li H., Wang H. (2019). Evaluation and Optimization of Task-oriented Measurement Uncertainty for Coordinate Measuring Machines Based on Geometrical Product Specifications. Appl. Sci..

[23] Vrba I., Palencar R., Hadzistevic M., Strbac B., Spasic-Jokic V., Hodolic J. (2015). Different Approaches in Uncertainty Evaluation for Measurement of Complex Surfaces Using Coordinate Measuring Machine. Meas. Sci. Rev..

[24] Kubátová D., Melichar M., Kutlwašer J. (2017). Evaluation of Repeatability and reproducibility of CMM equipment. Procedia Manuf..

[25] Wojtyła M., Rosner P., Płowucha W., Forbes A.B., Savio E., Balsamo A. (2022). Validation of the sensitivity analysis method of coordinate measurement uncertainty evaluation. Measurement.

[26] Stojadinovic S.M., Majstorovic V.D., Durakbasa N.M., Stanic D. (2022). Contribution to the development of a digital twin based on CMM to support the inspection process. Meas. Sens..

[27] Ren G., Qu X., Chen X. (2020). Performance Evaluation and Compensation Method of Trigger Probes in Measurement Based on the Abbé Principle. Sensors.

[28] Mazur T., Rucki M., Jakubowicz M., Cepova L., Diering M., Wieczorowski M., Harugade M., Pereira A. (2022). Analysis of the Direction-Dependent Point Identification Accuracy in CMM Measurement. Advances in Manufacturing III. Manufacturing 2022.

[29] McGarry L., Butterfield J., Murphy A. (2022). Assessment of ISO Standardisation to Identify an Industrial Robot’s Base Frame. Robot. Comput.-Integr. Manuf..

[30] Wan N., Zhuang Q., Guo Y., Chen Z.C., Fang Z. (2022). A new stylus orientation planning strategy for sculpture surface inspection based on touch position graph. Measurement.

[31] Mazur T., Rucki M., Gutsalenko Y. (2021). Accuracy analysis of the curved profile measurement with CMM: A case study. FACTA Univ. Ser. Mech. Eng..

[32] Li Y., Zeng L., Tang K., Li S. (2019). A dynamic pretravel error prediction model for the kinematic touch trigger probe. Measurement.

